# LPS stimulation of purified human platelets is partly dependent on plasma soluble CD14 to secrete their main secreted product, soluble-CD40-Ligand

**DOI:** 10.1186/s12865-015-0067-2

**Published:** 2015-01-31

**Authors:** Pauline Damien, Fabrice Cognasse, Marie-Ange Eyraud, Charles-Antoine Arthaud, Bruno Pozzetto, Olivier Garraud, Hind Hamzeh-Cognasse

**Affiliations:** Université de Lyon, GIMAP-EA3064, 15 rue Ambroise Paré, 42023 Saint-Etienne, France; EFS Auvergne-Loire, Saint-Etienne, France; Institut National de la Transfusion Sanguine, Paris, France

**Keywords:** Cytokine, Inflammation, Lipopolysaccharide, Platelet, Soluble CD14, TLR4

## Abstract

**Background:**

Platelets are instrumental to primary haemostasis; in addition, as they are central to endothelium vascular repair, they play a role in physiological inflammation. Platelets have also been demonstrated to be key players in innate immunity and inflammation, expressing Toll-like receptors (TLRs) to sense microbial infection and initiate inflammatory responses. They are equipped to decipher distinct signals, to use alternate pathways of signalling through a complete signalosome, despite their lack of a nucleus, and to adjust the innate immune response appropriately for pathogens exhibiting different types of ‘danger’ signals. Previous work has described the two main LPS isoforms-TLR4 activation pathways in purified platelets. However, the precise mechanism of TLR4 signalling in platelets is not completely unravelled, especially how this signalling may occur since platelets do not express CD14, the TLR4 pathophysiological companion for LPS sensing. Thus, we investigated from what source the CD14 molecules required for TLR4 signalling in platelets could come.

**Results:**

Here we show that CD14, required for optimal response to LPS stimulation, is obtained from plasma, but used with restrictive regulation. These data add to the body of evidence that platelets are closer to regulatory cells than to first line defenders. The readout of our experiments is the canonical secreted cytokine-like protein, soluble (s)CD40L, a molecule that is central in physiology and pathology and that is abundantly secreted by platelets from the alpha-granules upon stimulation.

**Conclusions:**

We show that sCD14 from plasma contributes to LPS/TLR4 signalling in platelets to allow significant release of soluble CD40L, thereby elucidating the mechanism of LPS-induced platelet responses and providing new insights for reducing LPS toxicity in the circulation.

## Background

The principal role of platelets is to control primary haemostasis. Physiologically, they patrol the large vascular system, detect endothelium insults and repair vascular damage to prevent leakage [1] To this end, platelets are equipped with molecular detectors that sense molecules abnormally exposed in the vessels, indicating alterations, i.e., danger [2]. It has been postulated that tissue repair, beginning with danger exclusion and healing, is important for the initiation of inflammation, especially subsequent to ‘danger’ signalling [3], indicating a possible role for platelets in this process [4]. Supporting this hypothesis is the discovery that platelets intervene throughout the spectrum of tissue physiology and pathology/inflammation: this has been exemplified in transfusion hazards [5] but also, importantly, in organ inflammatory pathology [6,7].

For many decades, it has been known that platelets can engulf infectious pathogens, though the nature of binding and endocytosis as well as the outcome of the pathogen within the platelet is still largely unknown. There is now a large body of evidence that platelets are intimately linked with infection. This arises from clinical observations of bacteraemia and sepsis, situations where there is usually a platelet drop and a risk of bleeding, and from experimental animal models. Numerous factors have been considered, outlining the complexity of this physiopathology [8].

Common to all platelet functions *in vivo*, including primary haemostasis, physiological inflammation and pathogen sensing, and leading to a wide range of outcomes, from the possible clearance of pathogens to acute pathology, is the discrete secretion of factors. There are three sources of platelet associated molecules: the alpha-granules, the dense granules, and cleavage from membranes. Platelet associated membrane molecules are either endogenous, or absorbed from the environment (principally the plasma) [9]. Platelets can also secrete and re-internalize some of their products, making their physiology much more complex than initially thought [10]. Despite being non-nucleated, platelets can secrete enormous amounts of cytokines and biological response modifiers [1,4], depending on the nature of the stimuli applied to the cells [11]. A call for a complete revisit of the interactions between platelets and bacteria resulted from reports that platelets express several types of pathogen recognition receptors, including Toll-like receptors (TLRs), both in human and mouse [12,13]. However, there may be phenotypic and fundamental functional differences between the expression of TLRs in mouse versus human platelets. Furthermore, a signalosome was described in platelets, with distinct signalling pathways, through NF-κB activation [14,15].

In humans, platelets bind different types of Gram-negative bacteria lipopolysaccharide (LPS) via membrane-expressed TLR4, and can sense them differentially and secrete discrete cytokine profiles [11,16]. In mice, although there is clear evidence of LPS-TLR4 binding, subsequent platelet activation and cytokine secretion remains largely unclear. The question as to whether LPS-TLR4 binding can lead to the secretion of soluble CD40 ligand (sCD40L), one of the major platelet-secreted factors, has been recently addressed [17]; the possibility that genetic polymorphisms explain the variations in affinity or interactions with physiologically relevant molecules has been raised.

However, another puzzling issue remains regarding LPS-TLR4 binding and functioning: the interplay with lipopolysaccharide binding protein (LBP) and CD14, considered obligate partners of TLR4 for ligation of LPS. This LPS-TLR4 signalling pathway has been dissected in the monocyte system, which is extremely responsive to LPS in both mouse and human systems [18]; but in contrast to monocytes, platelets do not express CD14 [19,20].

In the present study, we tested the hypothesis that human platelets can “borrow” soluble CD14 (sCD14) from the plasma environment to activate membrane TLR4 signalling when exposed to LPS. These studies will increase our understanding of how platelets function in pathology in infectious situations, and may lead to medical interventions to prevent either the risk of bleeding or of forming microthrombi, both dangerous conditions in patients.

## Methods

### Platelet preparation

In accordance with French regulations (French Public Health Code, Article L. 1223–3), platelets were obtained from regular blood donors who signed a form, after being informed, indicating that they do not preclude the use of their sample for medical research. Therefore, by signing the donation form, blood donors give their consent to take part in the study. Peripheral blood was collected from healthy donors in endotoxin-free tubes with 3.2% sodium citrate (Vacutainer®, Becton Dickinson, San Jose, CA, USA).Platelet-rich plasma (PRP) was prepared by centrifugation at 150 × *g* for 12 min at 22°C. To assess the quality of PRP preparation, leukocytes were also recovered at the interphase between the PRP and the red cell pellet. Red cells contaminating the leukocyte suspension were removed with RBC lysis buffer, according to the manufacturer’s instructions (eBioscience-Affimetrix, Paris, France). For some experiments, platelets from PRP were washed twice in PBS and resuspended in Tyrode’s buffer (Sigma Aldrich, Lyon, France). The mean leukocyte contamination of the PRP preparation was 0.18% of total cells. Analysis of cells showed no expression of CD14, CD15 and CD19 but a significant expression of CD3, suggesting that the contaminating cells were mostly T cells and not monocytes.

### Flow cytometry

CD63 expression, characteristic of activated platelets, was measured in PRP by flow cytometry after gating on CD41-positive events (CD41 is specifically expressed by platelets) in the R5 region of the cytogram, as described previously [11]. In the same way, CD3, CD14, CD15 and CD19 lineage marker expression was assessed by immunostaining platelets with anti-human monoclonal antibodies (mAbs). Leukocytes obtained after PRP preparation and red cell lysis underwent the same staining procedure and were analysed by flow cytometry in the R6 region of the cytogram.

Phycoerythrin-conjugated mAbs against human CD63 (clone H5C6), allophycocyanin-conjugated mAbs anti-human CD41 (clone HIP8) and fluorescein isothiocyanate (FITC)-conjugated mAbs against human CD3 (clone HIT3a), CD14 (clone M5E2), CD15 (clone HI 98) or CD19 (clone SJ25CI) were purchased from BD Biosciences (Le Pont de Claix, France). Positive events were recorded by FACSCalibur (BD Biosciences) and analysed with Cellquest Pro (BD Biosciences).

### Platelet stimulation

Platelets (3 × 10^8^) were stimulated for 30 min at room temperature (RT) with ultrapure S-LPS from *E. coli* 0111: B4 (1–10 μg/ml; Cayla-Invivogen, Toulouse, France) and recombinant CD14 (0.25 μg/ml; R&D Systems Europe Ltd, Lille, France). Thrombin receptor activator peptide (TRAP; 50 μg/ml; Sigma-Aldrich, Saint Quentin-Fallavier, France) was used as a positive control. Platelets were exposed to LPS for 30 min in the presence or absence of mouse anti-human CD14 (clone 18D11; F(ab′)2; 20 μg/ml; LifeSpan Biosciences, Inc., Seattle, WA, USA) or the isotype control F(ab′)2 (clone 15H6; 20 μg/ml; LifeSpan Biosciences).

### sCD14 depletion in plasma

Anti-CD14 magnetic beads (Dynal, Life Technologies, Saint Aubin, France) were used to capture sCD14 molecules by negative selection according to the manufacturer’s protocols defined for immunoprecipitation. Briefly, 1500 μl of Dynabeads were added to 450 μl of PRP, incubated 1 hour at RT away from light and removed from PRP using a magnetic particle concentrator.

### Stimulation readout: platelet secretion of sCD40 ligand

The levels of sCD40L were measured in triplicate in aliquots of unstimulated (control), TRAP- or TLR4 ligand-stimulated platelet supernatants by enzyme-linked immunoabsorbent assay (ELISA; Abcys, Paris, France); details have been provided previously [11,21]. Absorbance at 450 nm was measured by ELISA reader (Multiskan EX; Labsystem, Helsinki, Finland).

### Statistical analysis

The Wilcoxon paired test was used for comparison of percentages of activated cells and of sCD40L secretion in the different experiments. Values are expressed as mean ± SD. A *P* value of <0.05 was considered statistically significant.

## Results

### Cell studies

**Figure 1:**
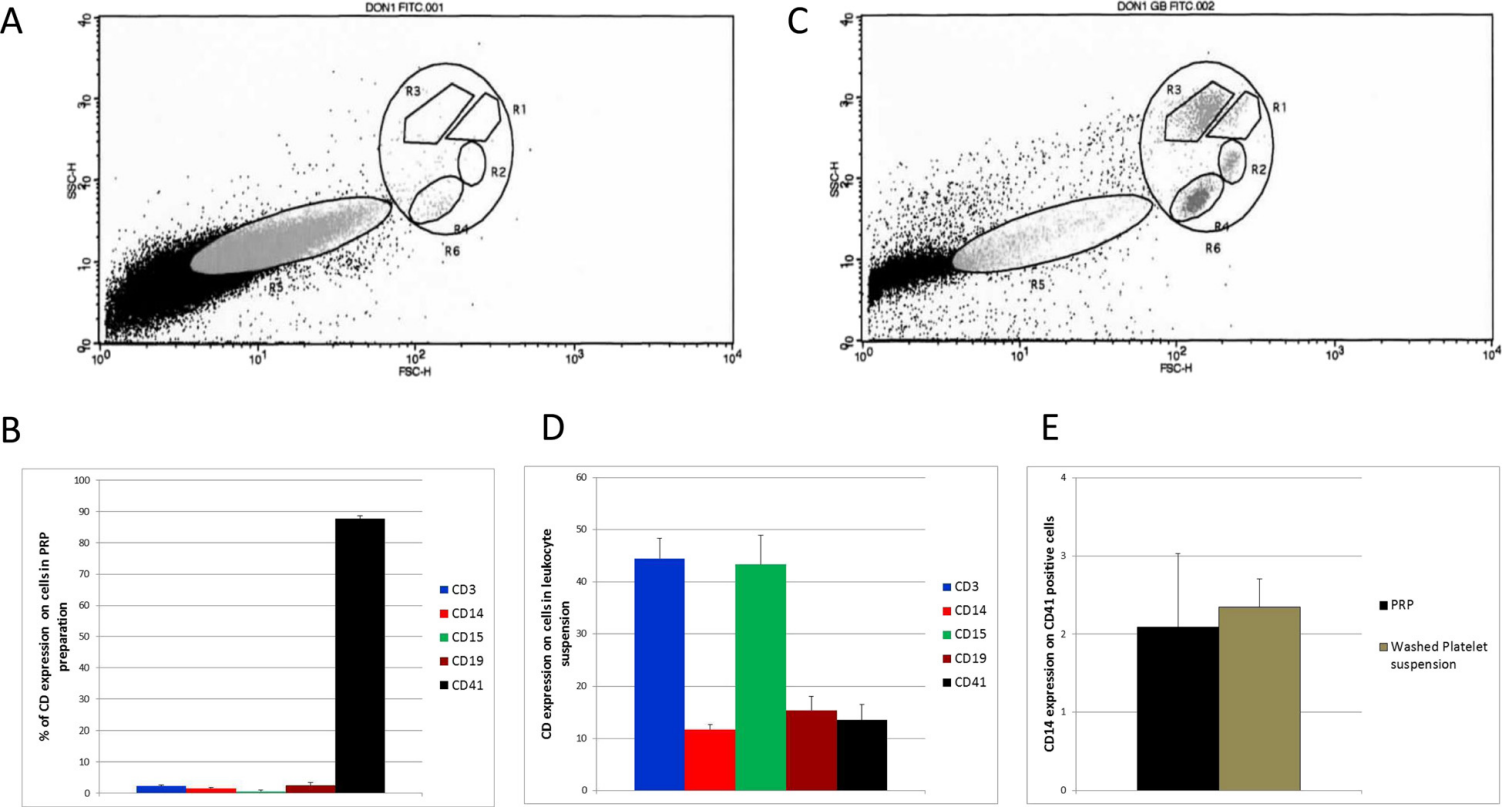
**Flow cytometry analysis of CD3, CD14, CD15, CD19, and CD41 expression.** Platelet-rich plasma (PRP) was prepared by centrifugating peripheral blood from healthy donors and collecting in the upper phase. Leukocytes were also recovered at the interphase between the PRP and the red cells. The contaminating red cells in the leukocyte suspension were removed by lysis. Platelets **(A,D)** and leukocytes **(C,D)** were stained for lineage markers (CD3^+^ T cells, CD14^+^ monocytes, CD15^+^ neutrophils, CD19^+^ B cells and CD41+ platelets). Fluorescence analysis was gated on the R5 region for PRP **(A,B)** and on R6 region of the cytogram for leukocytes **(C,D)**. The presence of mononuclear cells in PRP preparation, although significant, was clearly lower than in leukocyte preparation. The expression of CD14 on platelets from PRP was compared to that of washed platelets **(E)** after gating on CD41-positive cells in the R5 region of the cytogram (data are expressed as percentage of CD expression ± SD; n = 10 experiments). Cytograms shown **(A,C)** are representative of 10 independent experiments. *P < 0.05 (Wilcoxon paired test; platelets PRP vs. washed platelet suspension).

The PRP composition has been analysed for its purity and representative pictures are shown in Figure 1. Of particular note, we have focused on the CD14^+^ and CD41^+^CD14^+^ populations (CD41 is a platelet marker). Neither PRP nor washed cell preparations contain significant CD14^+^-expressing cells; a 2% contamination or expression appears constant in this series of experiments (Figure 1E).

### LPS-TLR4 activation studies: what role for exogenous CD14?

**Figure 2:**
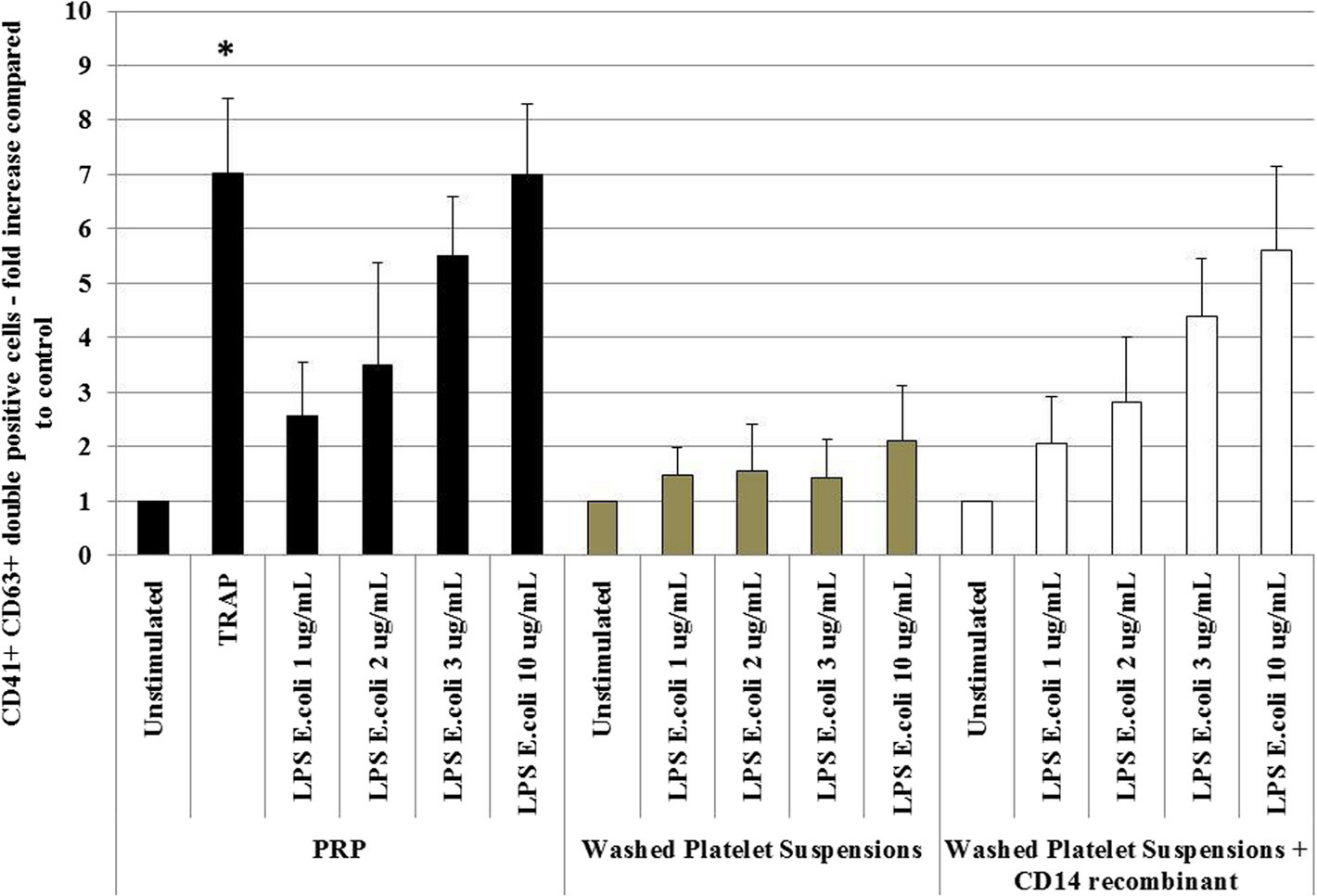
**Flow cytometry analysis of CD63 expression on CD41** ^**+**^ **platelets.** Platelets were or stimulated or not with TRAP (50 μg/ml, 30 min at RT). Platelets from platelet-rich plasma (PRP; black bars), washed (PBS) platelet suspensions (khaki bars) or washed (PBS) platelet suspensions with added recombinant soluble CD14 (white bars) were tested. Platelets were then stimulated, or not, with *E. coli* LPS (1–10 μg/ml; 30 min at RT), stained with allophycocyanin-conjugated anti-CD41 and phycoerythrin-conjugated anti-CD63 mAbs. Gating was made on the platelet population, (R5 region according to FSC/SSC comparable to the Figure 1 cytogram). Double positive cells (CD41 + CD63+) were then measured. Data are expressed as fold increase of controls (± SD; n = 10 independent experiments). *P < 0.05 (Wilcoxon paired test; stimulated vs. non-stimulated).

Surface expression of CD63, a characteristic marker of platelet activation, increased 7-fold upon the addition of thrombin receptor activator peptide (TRAP) Ser-Phe-Leu-Leu-Arg-Asn-Pro-Asn-Asp-Lys-Tyr-Glu-Pro-Phe – TRAP 14) (50 μg/ml; 30 min), which was used as an activation control (Figure 2), and 5.5-fold upon exposure to *E. coli* LPS for 30 min (≥3 μg/ml; Figure 2; *P* < 0.05). This activation was observed in the presence of autologous plasma rich medium (sCD14 concentration: 24.90 ± 7.2 ng/ml), and was lost after platelet washing (residual sCD14 concentration: 2.79 ± 1.47 ng/ml). Activation was partially restored upon addition of exogenous, recombinant CD14 (0.25 μg/ml; Figure 2), indicating that CD14 is required to achieve activation by LPS through TLR4.

### Consequence of platelet stimulation by LPS in the presence or absence of sCD14 on sCD40L secretion

**Figure 3:**
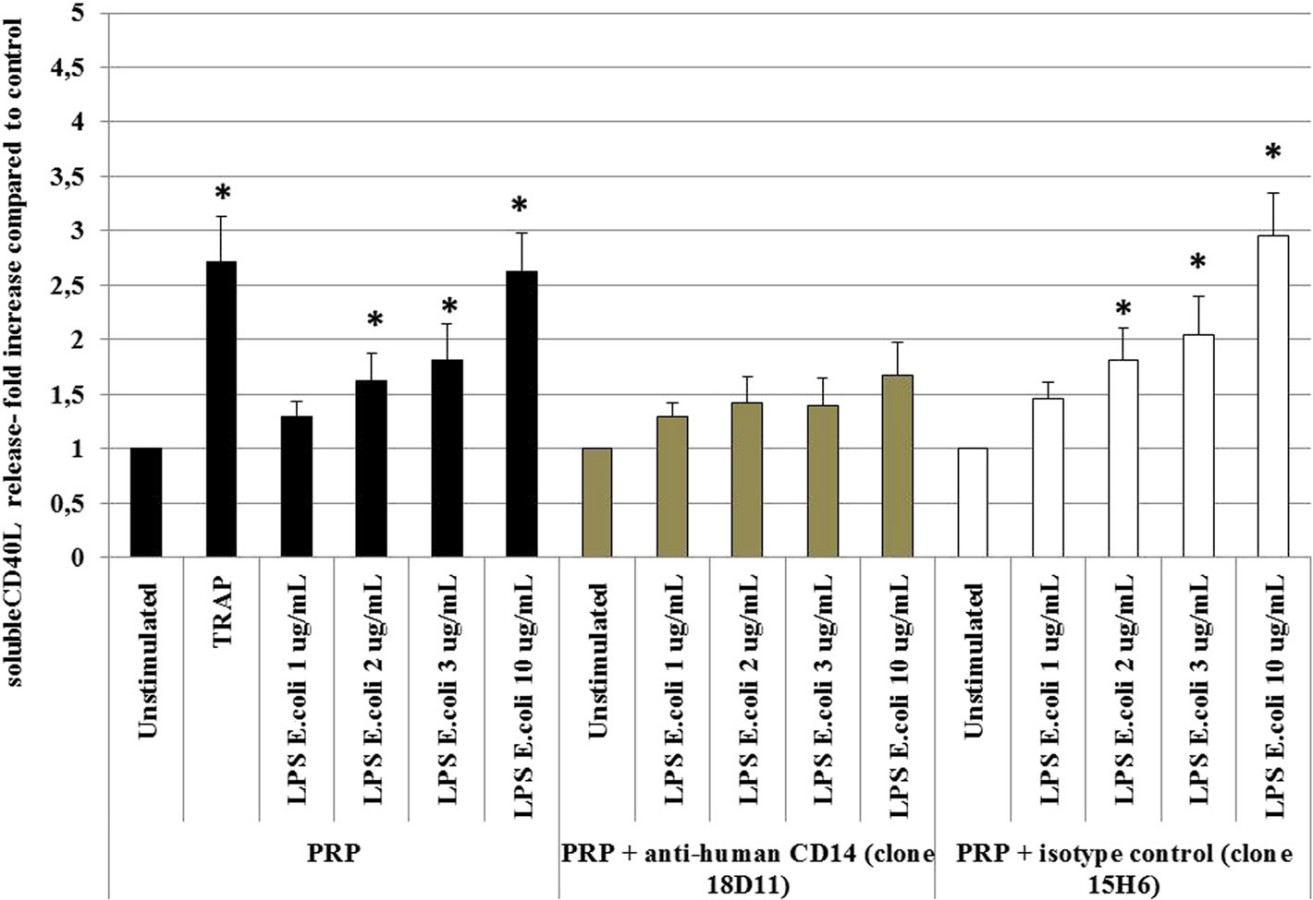
**Platelet sCD40L production and secretion.** Platelets were unstimulated or stimulated with TRAP (50 μg/ml, 30 min at RT). Platelets from platelet-rich plasma (PRP; black bars), or from platelet suspensions with added anti-human CD14 (clone 18D11 20 μg/ml; grey bars) or isotype control (clone 15H6; white bars) were tested. Platelets were unstimulated or stimulated with *E. coli* LPS (1–10 μg/ml; 30 min at RT). Mean sCD40L release was 1.2 ng/ml for unstimulated PRP and 1.5, 1.9, 2.2 and 3.1 ng/ml for LPS stimulation at concentrations of 1, 2, 3 and 10 μg/ml, respectively. Data are expressed as fold increase of controls (± SD; n = 10 independent experiments). *P < 0.05 (Wilcoxon paired test; stimulated vs. non-stimulated).

Exposure to TRAP (50 μg/ml; 30 min) increased sCD40L secretion levels measured in the supernatant 2.7-fold (Figure 3). Stimulation of platelets in their plasma environment with 2, 3 or 10 μg/ml LPS caused an increase in sCD40L secretion of 1.62-, 1.82- or 2.63-fold, respectively (Figure 3; *P* < 0.05). However, LPS when used at a 1 μg/ml concentration failed to induce sCD40L release by platelets.

To test the hypothesis that plasma-derived CD14 is involved in LPS-TLR4-induced signalling leading to cytokine secretion, we sought to use an anti-CD14 neutralizing antibody to block CD14. Anti-CD14 antibody (20 μg/ml) significantly inhibited the augmentation of platelet sCD40L secretion upon LPS stimulation (Figure 3).

As an additional control and to confirm the role of plasma sCD14, we attempted to deplete sCD14 from plasma before reusing it with autologous platelets for LPS-TLR4 stimulation. To this end, we used anti-CD14 beads (Dynal, Life Technologies SAS, Saint Aubin, France). However, despite a significant decrease of sCD14 in plasma (not shown), this was not sufficient to test whether sCD14-substantially depleted plasma renders the LPS-TLR4 unstable for ensuing signalling.

## Discussion

It has been proposed that, to achieve activation by LPS, CD14 association with platelet TLR4 is necessary [11]. This is the accepted model as described in the monocyte, the dominant target of LPS sensing. Human dendritic cells also signal through this pathway. As platelets are known to display functional TLR4 and react to LPS-TLR4 engagement [13,22-24] but do not constitutively express CD14, two plausible explanations can be considered: CD14 is indeed used by platelets but comes from an external source, or TLR4/LBP/MD2 uses another stabilizer for LPS sensing through TLR4. The present study shows that sCD14 is necessary for platelet responses to LPS, particularly for the release of sCD40L, the dominant platelet-secreted factor. Platelets are the main source of sCD40L [25]. sCD40L is a master inflammatory cytokine in many systems, in particular for the production of atheroma plaques and inflammation in cardiovascular disease [1].

MD2 is a glycoprotein (20–25 kDa) expressed as both a TLR4-bound complex on cell membranes and as an active soluble form (sMD2) in the extracellular medium. Soluble MD2 might increase and modulate steady state levels of TLR4 on the surface of platelets and thus have a role in innate immunity. Indeed, in eukaryotic cells, LPS is transferred from CD14 to a heterodimeric protein complex involving TLR4 and the accessory molecule MD2, which forms a complete recognition site for LPS. Lauer et al. [26] showed that LPS responsiveness in epithelial cells can be controlled by circulating sMD2 that gains access to extravascular spaces with endogenous TLR4 on the cell membrane. sMD2 does not appear to modulate TLR4 gene expression directly and TLR4 mRNA levels did not significantly change in response to incubation with sMD2. In accordance with Zhang G et al., [22] we observed the expression of platelet MD2/TLR4 as 9.87 ± 2.48%. Even though we observed a significant (*P* < 0.05) increase after TRAP stimulation (23.92 ± 6.67%), we did not observe significant modulation after *E. coli* LPS incubation (6.58 ± 1.19%; data not shown), nor CD14 on platelet membrane. This discrepancy with the observation of Zhang et al. could be explained by the immunostaining technique used. Indeed, while we stained platelets directly with a specific anti-CD14 FITC-conjugated mAb (clone M5E2, BD Bioscience) Zhang et al. used indirect immunostaining with a mAb against human CD14 (clone UCH-M1) and a FITC-conjugated mouse IgG.

We thus extended previous findings in showing the requirement of sCD14 on platelets as well, but in contrast to monocytes, platelets have to obtain it from the circulation. This could be modelled by addition of recombinant sCD14 to purified platelets exposed to LPS; and this could be partly neutralized in the additional presence of neutralizing Abs. Full activation or suppression of responses was not observed in these experiments, probably because the reagents (sCD14, LPS, mAb) used do not exactly mimic the endogenous folding and presentation of components where circulating platelets in the blood flow encounter a LPS-harbouring live bacterium. However, the model provides interesting clues to the physiological functioning of platelets in septic inflammation. Despite many attempts, it was not possible to completely deplete sCD14 from plasma to provide further confirmation of its requirement in LPS-TLR4 signalling. Again, we propose that the folding, stability or even purity of the commercial reagent was not optimal to allow any firm conclusion, but the partial demonstration strengthens the proposed model. It cannot, however, be ruled out that another, as-yet undefined, plasma factor is used by platelets to mimic CD14 within the TLR4/LBP/TLR2 complex.

## Conclusions

Dissecting the pathways of platelet physiology (primary haemostasis), but also of physiopathology (sepsis, cardiovascular diseases, systemic inflammation, autoimmunity, transfusion and transplantation pathology) [1,4,27] is valuable for developing new drugs or treatments, taking advantage of the benefits of platelets while removing their deleterious effects, especially in patients with severe conditions. The contribution of absorbed sCD14 on platelets to complement LPS/TLR4 signalling and the subsequent production of proinflammatory products increase our understanding of platelet activation by bacterial products, and may provide a new direction for treatment of patients with bacteraemia/sepsis. Controlling LPS-induced responses by circulating platelets, as well as leukocytes, could thus reduce systemic LPS toxicity and inflammation related to microbial invasion.

## Abbreviations

FITC: Fluorescein isothiocyanate
LBP: Lipopolysaccharide binding protein
LPS: Lipopolysaccharide
mAb: Monoclonal antibodies
PRP: Platelet-rich plasma
RT: Room temperature
sCD14: Soluble CD14
sCD40L: Soluble CD40 ligand
sMD2: Soluble MD2
TLRs: Toll-like receptors
TRAP: Thrombin receptor activator peptide
